# Iodoarylation of Arylalkynes with Molecular Iodine in the Presence of Hypervalent Iodine Reagents

**DOI:** 10.3390/molecules14093132

**Published:** 2009-08-24

**Authors:** Md. Ataur Rahman, Tsugio Kitamura

**Affiliations:** Department of Chemistry and Applied Chemistry, Faculty of Science and Engineering, Saga University, Honjo-machi, Saga, 840-8502, Japan

**Keywords:** iodoarylation, alkyne, molecular iodine, hypervalent iodine, iodoethene

## Abstract

Iodoarylation of arylacetylenes was performed using a simple reagent system composed of molecular iodine and [bis(benzoyloxy)iodo]benzene. Most arylacetylenes efficiently underwent the iodoarylation reaction with electron-rich arenes to give *trans* 1,1-diaryl-2-iodoethene adducts regio- and stereoselectively. As an exception, the iodoarylation of *p*-methoxyphenylacetylene resulted in a mixture of *E*- and *Z*-isomers of the corresponding product.

## 1. Introduction

Alkenyl halides, especially iodides, are synthetically useful compounds and have attracted considerable attention of organic chemists because the iodo group is useful for further elaboration by subjecting the iodoalkene units to metal-catalyzed cross-coupling reactions [1,2,3]. However, direct iodination of alkenes to iodoalkanes using molecular iodine (I_2_) is generally impractical because of the low reactivity of iodine.

We have found that the iodination reaction of aromatic compounds with I_2_ proceeds smoothly in the presence of potassium peroxodisulfate (K_2_S_2_O_8_) as an oxidant to give iodoarenes in good yields [4]. To further explore the use of I_2_, we extended the iodination reaction of arenes to iodoarylation of alkynes with arenes. It was assumed that an alkyne would be activated by iodine to form a bridged iodonium ion which would then undergo electrophilic substitution with an arene. However, there are no reports in which molecular iodine is used as an iodine source for the iodoarylation of alkynes. Recently, Barluenga *et al*. reported the iodoarylation of arenes using bis(pyridine)iodonium(I) tetrafluroborate as an iodine source [5]. This reagent is commercially available, but very expensive, and its preparation requires the use of toxic mercury oxide [6]. Therefore, a convenient, safe, and simple iodine reagent for the iodoarylation of alkynes is strongly required. Preliminarily, we have found that the iodoarylation of alkynes proceeds effectively in the presence of a simple reagent system: I_2_ and a hypervalent iodine species like PhI(OCOPh)_2_ [7]. Herein we report the details on the iodoarylation of alkynes with the reagent system consisting of molecular iodine and hypervalent iodine compounds. The results demonstrate a convenient and simple carbon-carbon bond formation process between electron rich arenes and aryl-substituted alkynes using molecular iodine in the presence of hypervalent iodine reagents.

## 2. Results and Discussion

**Scheme 1:**
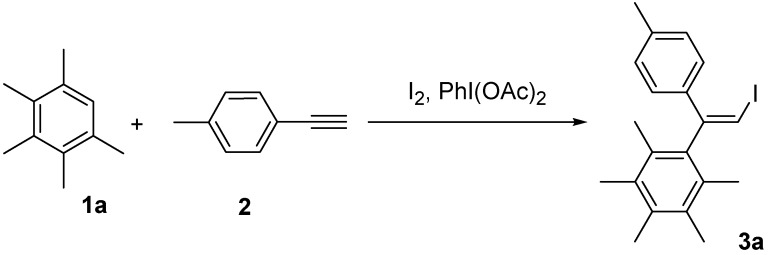
Reaction of alkyne **2** with arene **1a**.

Molecular iodine and hypervalent iodine compounds have witnessed a large growth in recent years [8,9,10,11,12,13,14,15,16,17,18,19,20]. In the present study we confined our attention to the formation of carbon-carbon bonds between arenes and aryl-substituted alkynes in the presence of molecular iodine and hypervalent iodine reagents. Initially, the present work concentrated on the efficiency of the formation of a carbon-carbon bond between pentamethylbenzene (**1a**) and *p*-methylphenylacetylene (**2**) in the presence of I_2_ and (diacetoxyiodo)benzene, PhI(OAc)_2 _(Scheme 1). PhI(OAc)_2 _is one of the most widely available commercial hypervalent iodine compounds and has been widely used in organic synthesis [8,9], making it the first choice among hypervalent iodine reagents tested .

### 2.1. Optimization of Reaction Conditions

To optimize the reaction conditions the reaction of **1a** and **2** using I_2_ in the presence of PhI(OAc)_2_ was investigated (Table 1). The iodoarylation reaction occurred in different solvent systems: 1,2-dichloroethane (DCE), AcOH, and MeCN to give 1-iodo-2-(4-methylphenyl)-2-(pentamethylphenyl)-ethene (**3a**; Entries 1-3). MeCN (Entry 3) gave the best results. Increasing the amount of **1a** resulted in a better yield of **3a** (Entry 4). Elevation of the reaction temperature also improved the yield of **3a** (Entry 5). The best overall result (78% yield) was obtained by the reaction at 82 ^o^C using 10 equivalents of **1a** (Entry 6). The reaction in EtOAc instead of MeCN resulted in a low yield (13%) of product **3a** (Entry 7).

**Table 1:** Optimization of reaction conditions for the reaction of **1a** and **2** in the presence of PhI(OAc)_2_.

Entry	1a (mmol)	PhI(OAc)_2_ (mmol)	Solvent (Amt.)	Temp. (^o^C)	Time (h)	Yield of 3a (%)^*^
1	1	1.25	DCE (2 mL)	45	28	12
2	5	3	AcOH (2 mL)	60	48	23
3	1.5	3	MeCN (2 mL)	60	48	31
4	5	3	MeCN (2 mL)	60	48	52
5	5	3	MeCN (4 mL)	78	56	67
6	10	3	MeCN (4 mL)	82	65	78
7	10	3	EtOAc (4 mL)	78	65	13

*Reaction conditions*: **1a**, **2** (1 mmol), I_2_ (1.25 mmol), PhI(OAc)_2_ and solvent. ^* ^Isolated yield based on **2**.

To improve the yield of iodoarylation product **3a**, we further examined the effect of different [bis(acyloxy)iodo]benzenes, PhI(OH)OTs, and AgOCOPh on the iodoarylation reaction. Several [bis(acyloxy)iodo]benzenes were prepared from benzoic acid, *m*- and *p*-chlorobenzoic acids, *p*-nitro-benzoic acid, *p*-toluic acid, and *p*-anisic acid, according to the literature method [21]. The results are given in Table 2. PhI(OCOPh)_2_ showed the highest activity among all other reagents, giving the product **3a** in 86% yield (Entries 1-6). [Bis(4-nitrobenzoyloxy)iodo]benzene also gave **3a** in good yield (80%), but we chose [bis(benzoyloxy)iodo]benzene as the activator because of the ready availability of benzoic acid. Koser’s salt, PhI(OH)OTs, and silver benzoate show little effect in the iodoarylation reaction (Entries 7 and 8).

**Table 2:** Effect of hypervalent iodine reagents on the reaction of **1a** with **2**.

Entry	Hypervalent iodine reagent	Yield of 3a (%)
1	PhI(OCOPh)_2 _	86
2	[Bis( *m*-chlorobenzoyloxy)iodo]benzene	78
3	[Bis( *p*-chlorobenzoyloxy)iodo]benzene	65
4	[Bis( *p*-nitrobenzoyloxy)iodo]benzene	80
5	[Bis( *p*-methylbenzoyloxy)iodo]benzene	74
6	[Bis( *p*-methoxybenzoyloxy)iodo]benzene	70
7	PhI(OH)OTs	33^a,b^
8	AgOCOPh	12^c^

*Reaction conditions*: **1a** (10 mmol), **2** (1 mmol), I_2 _(1.25 mmol), a hypervalent iodine regent (3 mmol), MeCN (6 mL), 82 °C, and 65 h. ^a ^Iodoarylation product **3a** was contaminated with hydroarylation product. ^b ^The reaction was conducted by using **1a** (3 mmol) in MeCN (4 mL) at 45 °C for 28 h. ^c ^**1a** (2 mmol), **2** (1 mmol), I_2_ (1 mmol), AgOCOPh (1 mmol), MeCN (2 mL), 40 °C and 36 h.

### 2.2. Scope of the iodoarylation reaction using I_2_ and PhI(OCOPh)_2_

**Scheme 2:**
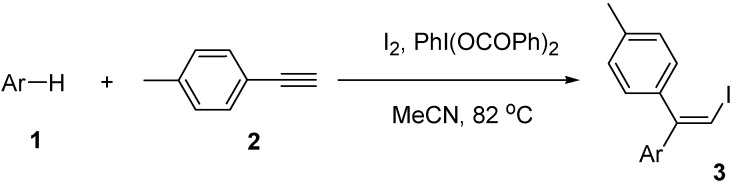
Reaction of alkyne **2** with arenes **1**.

The iodoarylation reaction of *p*-methylphenylacetylene (**2**) in the presence of PhI(OCOPh)_2_ was examined with different electron-rich arenes **1** (Scheme 2 and Table 3). The results of the reactions showed that the reaction with electron-rich arenes gave iodoarylation products in good yields. In particular the electron rich arene mesitylene (**1b**) gave iodoarylation product **3b** in high yield (Entry 1). The reaction with durene (**1c**) and bromomesitylene (**1d**) gave iodoarylation products **3c** and **3d** in 56 and 42% yields, respectively (Entries 2 and 3). The reaction with *p*-xylene (**1e**) gave a low yield of iodoarylation product **3e** (Entry 4).

**Table 3:** The reaction of alkyne **2** with arenes **1** in the presence of PhI(OCOPh)_2_.

Entry	Arene	Time (h)	Product	Isolated yield (%)
**1**	Mesitylene (**1b**)	65	**3b**	75
**2**	Durene (**1c**)	67	**3c**	56
**3**	Bromomesitylene (**1d**)	72	**3d**	42^a^
**4**	*p*-Xylene (**1e**)	72	**3e**	33^a^

*Reaction conditions*: Arene **1** (10 mmol), *p*-methylphenylacetylene (**2, **1 mmol), I_2 _(1.25 mmol), PhI(OCOPh)_2_ (3 mmol), MeCN (6 mL), and 82 °C.^ a ^A mixture of *E*- and *Z*-isomers.

**Scheme 3:**
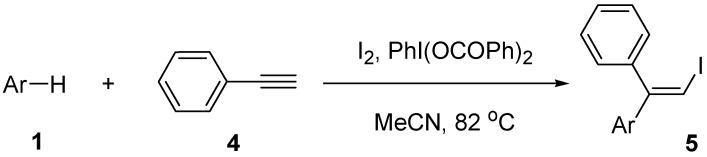
Reaction of alkyne **4** with arenes **1**.

Next, we examine the reaction of phenylacetylene (**4**) with different electron-rich arenes **1 **under the same reaction conditions as used for **2** (Scheme 3). The results are given in Table 4. The reaction of arenes such as **1a**-**1c** gave iodoarylation products **5a**-**5c** in good yields (Entries 1-3). Moderately activated arenes **1d** and **1e** showed a very low reactivity, giving low yields (32 and 24%) of iodoarylation products **5d** and **5e**, respectively.

**Table 4:** The reaction of alkyne **4** with arenes **1** in the presence of PhI(OCOPh)_2_.

Entry	Arene	Time (h)	Product	Isolated yield (%)
**1**	**1a**	65	**5a**	71
**2**	**1b**	70	**5b**	61
**3**	**1c**	73	**5c**	59
**4**	**1d**	76	**5d**	32
**5**	**1e**	72	**5e**	24

*Reaction conditions*: Arene **1** (10 mmol), phenylacetylene **4** (1 mmol), I_2 _(1.25 mmol), PhI(OCOPh)_2_ (3 mmol), and MeCN (6 mL) at 82 °C.

The iodoarylation reaction of arylacetylenes with arenes proceeded regio- and stereospecifically to give a single isomer of the possible *E*- and *Z*-1,1-diaryl-2-iodoethenes **3** and **5**. We attempted to determine the stereochemistry of iodoarylation product **3a** by NOE experiments, but only a small enhancement (3%) of the vinylic proton was observed when the *ortho* methyl proton was irradiated. This may be attributed to the deviation of the pentamethylphenyl ring from the olefinic plane. Next, we estimated the chemical shift of the vinylic proton according to the literature [22] and compared it with the observed value. The calculation of the chemical shift concerning the vinylic proton for iodoethenes **3** and **5** gives 6.51 ppm for the *E* isomer and 6.84 ppm for the *Z* isomer. In the ^1^H-NMR spectra of **3** and **5**, a singlet peak of the vinylic proton was observed at the range of 6.30–6.55 ppm. Therefore, the stereochemistry of iodoethenes **3** and **5** is considered to be *E*. This is in accord with the results obtained by Barluenga *et al*. [5].

**Scheme 4:**
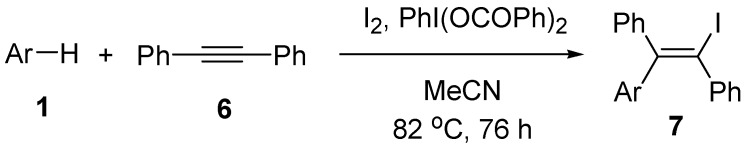
Reaction of alkyne **6** with arenes **1**.

The reaction of an internal alkyne, diphenylacetylene (**6**), with arenes **1** was further examined under the above reaction conditions (Scheme 4). The reaction of electron rich arenes such as **1a** and **1b** with **6** yielded the expected iodoarylation products **7a** and **7b**, but the yields were low (14 and 8%, respectively). The low yield of the reaction may be attributable to the low reactivity of diphenylacetylene.

**Scheme 5:**
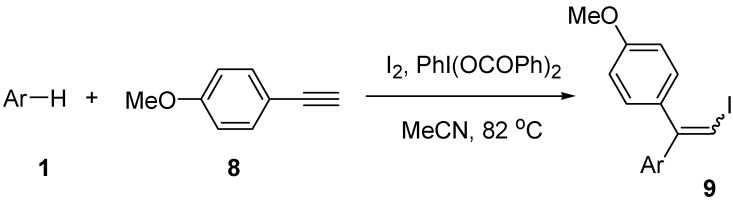
Reaction of alkyne **8** with arenes **1**.

Moreover, we examined the reaction of electron-rich *p*-methoxyphenylacetylene (**8**) with arenes **1** (Scheme 5). The results are shown in Table 5. The reaction of electron-rich arenes **1a-1c **gave iodoarylation products **9** in good yields (Entries 1-3). Moderately activated arenes such as **1d** and **1e** showed a low reactivity and the yields were 32 and 16%, respectively. In the case of arylacetylene **8**, the iodoarylation reaction lost its stereospecificity to give a mixture of *E*- and *Z*-isomers of iodoethenes **9**. This behavior is different from that observed in the cases of other arylacetylenes **2** and **4**.

**Table 5:** The reaction of alkyne **8** with arenes **1** in the presence of PhI(OCOPh)_2_.

Entry	Arene	Time (h)	Product^a^	Isolated yield (%)
1	**1a**	72	**9a**	75
2	**1b**	72	**9b**	63
3	**1c**	73	**9c**	62
4	**1d**	76	**9d**	32
5	**1e**	76	**9e**	16

*Reaction conditions*: Arene **1** (10 mmol), alkyne **8** (1 mmol), I_2_ (1.25mmol), PhI(OCOPh)_2_ (3 mmol), and MeCN (6 mL) at 82 °C. ^a ^Products **9** were obtained as a mixture of *E*- and *Z*-isomers.

Finally, we examine the reaction of *p*-fluorophenylacetylene (**11**) with arenes **1** under the above reaction conditions. The results are given in Table 6. The reaction with electron-rich arenes **1a-1c **gave iodoarylation products **12** in good yields (Entries 1-3). The similar reaction of **1d** and **1e** resulted in low yields (27 and 23%, respectively) of products **12**. Arylacetylene **11** showed a similar behavior to **2** and **4** to undergo stereospecific iodoarylation reaction.

**Scheme 6:**
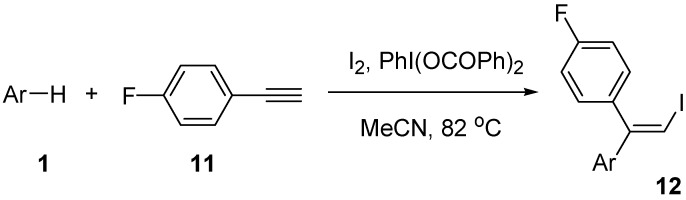
Reaction of alkyne **11** with arenes **1**.

**Table 6:** The reaction of alkyne **11** with arenes **1** in the presence of PhI(OCOPh)_2_.

Entry	ArH	Time (h)	Product	Isolated yield (%)
1	**1a**	72	**12a**	69
2	**1b**	72	**12b**	67
3	**1c**	73	**12c**	56
4	**1d**	76	**12d**	27
5	**1e**	76	**12e**	23

*Reaction conditions*: Arene **1** (10 mmol), *p*-fluorophenylacetylene **11** (1 mmol), I_2_ (1.25 mmol), PhI(OCOPh)_2_ (3 mmol), and MeCN (6 mL) at 82 °C.

No iodoarylation reaction occurred in the case of 1-hexyne and 3-butyn-2-one, suggesting that this iodoarylation reaction is applicable only to relatively electron-rich alkynes.

### 2.3. Mechanistic consideration

**Scheme 7:**
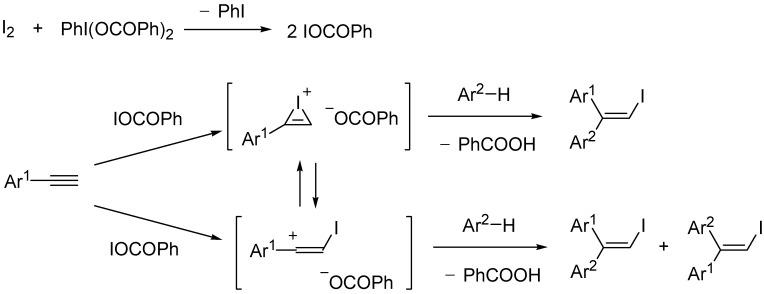
Proposed mechanism of iodoarylation reaction of alkynes.

The proposed mechanism of the iodoarylation reaction of arylacetylenes is shown in Scheme 7. Initially iodine reacts with PhI(OCOPh)_2_ to form a hypoiodite, IOCOPh [23,24], which actually undergoes iodination of an arylacetylene. The *in situ*-generated IOCOPh adds the arylacetylene to give a cyclic iodonium benzoate. In most cases, the cyclic iodonium form is stable compared with an open vinyl cation due to a significant neighboring participation of iodo group [25]. However, the open vinyl cation governs the reaction process in the case of *p*-methoxyphenylacetylene, where the vinyl cation is strongly stabilized by *p*-methoxyphenyl group and exists as the open form. The cyclic iodonium ion undergoes aromatic electrophilic substitution with an electron-rich arene to afford an iodoarylation product stereoselectively. The presence of the cyclic iodonium ion causes *trans* addition giving the stereochemically defined product. On the other hand, the open vinyl cation has a linear sp-hybridized structure and can react with an arene at both sides of the vacant p orbital. Accordingly, in the case of *p*-methoxyphenylacetylene, a mixture of *E*- and *Z*-isomers is formed. Benzoate anion can trap a proton generated by aromatic electrophilic substitution and prevent the reaction from occurring by protonation.

## 3. Conclusions

We have demonstrated that arylacetylenes undergo iodoarylation reaction in the presence of a simple reagent system composed of I_2_ and PhI(OCOPh)_2_. The iodoarylation reaction of most arylacetylenes with electron-rich arenes proceeds regio- and stereoselectively to give *trans* 1,1-diaryl-2-iodoethene adducts in good to high yields. In the case of *p*-methoxyphenylacetylene, the iodoarylation reaction affords a mixture of *E*- and *Z*-isomers of 1,1-diaryl-2-iodoethenes **9**. The procedure involves a simple and convenient reagent system and provides synthetically valuable iodoalkenes.

## 4. Experimental

### 4.1. General

All solvents and starting materials were used as received without further purification unless otherwise indicated. ^1^H-NMR (300 MHz) and ^13^C-NMR (75 MHz) were recorded on a JEOL JNM-Al 300 FT-NMR spectrometer in CDCl_3 _solution (TMS as an internal standard). Melting points were measured with a YANACO micro melting apparatus and are uncorrected. Column chromatographic separations were carried out using silica gel as the stationary phase. Pre-coated plates (silica gel 60 F_254_, MERCK) were used for TLC examination. All [bis(acyloxy)iodo]benzenes were prepared using a literature procedure [21]. Elemental analysis was performed by the Service Center of the Elementary Analysis of Organic Compounds, Faculty of Science, Kyushu University.

### 4.2. General procedure for the iodoarylation of alkynes

A mixture of an arene (10 mmol), an arylacetylene (1 mmol), I_2_ (1.25 mmol), PhI(OCOPh)_2_ (3 mmol) and MeCN (6 mL) was placed in a 25 mL round-bottom flask. The reaction mixture was stirred for about 5 min at room temperature and then refluxed at 82 ºC with stirring until the completion of the reaction. The reaction mixture was dissolved in CH_2_Cl_2_, (20 mL) and water (20 mL) was added to the CH_2_Cl_2_ solution. The mixture was then washed with 1M aqueous sodium thiosulphate solution to remove the unreacted iodine. The aqueous reaction mixture was extracted with CH_2_Cl_2_ (4 × 10 mL) and the CH_2_Cl_2_ extract was dried over anhydrous sodium sulfate. Finally, the solvent was removed under reduced pressure below 40 ºC. Individual pure compounds were isolated from the reaction mixture by column chromatography on silica gel using hexane and CH_2_Cl_2_ as eluent. Data of 1,1-diaryl-2-iodoethenes **3a-e**, **5a-e**, and **12a-e** were previously reported in the literature [7].

*1-Iodo-2-(pentamethylphenyl)-1,2-diphenylethene* (**7a**): Pale yellow crystalline solid; mp 194.5-197 °C; ^1^H-NMR δ: 7.44-6.99 (m, 10H, ArH), 2.20 (s, 6H, 2xMe), 2.08 (s, 3H, Me), 2.03 (s, 6H, 2xMe); ^13^C-NMR δ: 149.34, 144.94, 144.66, 137.66, 133.88, 132.35, 130.48, 129.37, 128.35, 127.64, 127.40, 127.31, 100.50, 18.80, 16.67, 16.49 (one peak overlapped); Anal. calcd. for C_25_H_25_I: C, 66.38; H, 5.57. Found: C, 66.34, H, 5.59.

*1-Iodo-1,2-diphenyl-2-(2,4,6-trimethylphenyl)ethene* (**7b**): Pale yellow crystalline solid; mp 154-156 °C; ^1^H-NMR δ: 7.42-7.06 (m, 10H, ArH), 6.64 (s, 2H, ArH), 2.16 (s, 6H, 2x Me), 2.13 (s, 3H, Me); ^13^C-NMR δ: 147.55, 144.50, 144.19, 137.29, 136.77, 135.40, 129.52, 128.93, 128.39, 127.66, 127.60, 127.56, 127.42, 101.39, 20.92, 20.75; HRMS-EI: *m*/*z* calcd. for C_23_H_21_I [M]^+^: 424.0688; found: 424.0689.

*1-Iodo-2-(4-methoxyphenyl)-2-(pentamethylphenyl)ethene* (**9a**): Pale yellow crystalline solid; mp 110-111.5 °C; a mixture of *E*- and *Z*-isomers (92:8); ^1^H-NMR δ: 7.42 (d, *J* = 9.0 Hz, ArH), 7.14 (d, *J* = 9.0 Hz, ArH), 7.02 (s, =CH), 6.82 (d, *J* = 9.0 Hz, ArH), 6.76 (d, *J* = 9.0 Hz, ArH), 6.24 (s, =CH), 3.78 (s, OMe), 3.76 (s, OMe), 2.28 (s, Me), 2.23 (s, 2xMe), 2.19 (s, 2xMe), 2.14 (s, Me), 2.04 (s, 2xMe); ^13^C- NMR δ: 159.29, 159.01, 152.24, 150.80, 139.69, 138.94, 134.20, 132.68, 132.46, 132.09, 131.34, 130.45, 130.30, 130.18, 129.52, 127.65, 113.84, 113.07, 79.28, 75.80, 55.20, 55.14, 18.06, 17.60, 16.94, 16.89, 16.80, 16.58; HRMS-EI: *m*/*z* calcd. for C_20_H_23_IO [M]^+^: 406.0794; found: 406.0791.

*1-Iodo-2-(4-methoxyphenyl)-2-(2,4,6-trimethylphenyl)ethene* (**9b**): Pale yellow highly viscous liquid; a mixture of *E*- and *Z*-isomers (87:13); ^1^H-NMR δ: 7.36-6.76 (m, ArH and =CH), 3.77 (s, 2xOMe), 2.33 (s, Me), 2.27 (s, Me), 2.21 (s, 2xMe), 2.06 (s, 2xMe); HRMS-EI: *m*/*z* calcd. for C_18_H_19_IO [M]^+^: 378.0481; found: 378.0479.

*1-Iodo-2-(4-methoxyphenyl)-2-(2,3,5,6-tetramethylphenyl)ethene* (**9c**): Pale yellow crystalline solid; mp 108-110 °C; a mixture of *E*- and *Z*-isomers (51:49); ^1^H-NMR δ: 7.36-6.77 ( m, ArH and =CH), 3.78 (s, 2xOMe), 2.25 (s, 2xMe), 2.21 (s, 2xMe), 2.14 (s, 2xMe), 1.99 (s, 2xMe); ^13^C-NMR δ: 159.37, 159.06, 157.94, 151.51, 144.75, 141.42, 134.16, 133.68, 133.40, 131.69, 131.05, 130.91, 130.83, 130.51, 130.15, 127.59, 113.89, 113.12, 79.04, 55.22, 55.16, 20.12, 20.11, 16.47, 15.82; HRMS-EI: *m*/*z* calcd. for C_19_H_21_IO [M]^+^: 392.0637; found: 392.0637.

*1-(3-Bromo-2,4,6-trimethylphenyl)-2-iodo-1-(4-methoxyphenyl)ethene* (**9d**): Pale yellow highly viscous liquid; a mixture of *E*- and *Z*-isomers (58:42); ^1^H-NMR δ: 7.81-6.68 ( m, ArH and =CH), 3.86 (s, OMe), 3.79 (s, OMe), 2.45 (s, Me), 2.38 (s, Me), 2.37 (s, Me), 2.21 (s, Me), 2.16 (s, Me), 2.01 (s, Me); HRMS-EI: *m*/*z* calcd. for C_18_H_18_BrIO [M]^+^: 455.9586; found: 455.9583.

*1-(2,5-Dimethylphenyl)-2-iodo-1-(4-methoxyphenyl)ethene* (**9e**): Pale yellow highly viscous liquid; a mixture of *E*- and *Z*-isomers (60:40), ^1^H-NMR δ: 7.76-6.73 (m, ArH and =CH), 3.86 (s, OMe), 3.79 (s, OMe), 2.33 (s, Me), 2.32 (s, Me), 2.21 (s, 2xMe); ^13^C-NMR δ: 159.08, 157.55, 157.22, 144.44, 143.91, 139.27, 136.44,135.76, 131.30, 130.55, 130.43, 129.98, 129.93, 129.02, 128.78, 128.39, 113.40, 110.02, 85.30, 77.21, 56.33, 55.20, 20.97, 19.14, 19.09, 17.74; HRMS-EI: *m*/*z* calcd. for C_17_H_17_IO [M]^+^: 364.0324; found: 364.0327.
